# Serum sodium based modification of the MELD does not improve prediction of outcome in acute liver failure

**DOI:** 10.1186/1471-230X-13-58

**Published:** 2013-04-03

**Authors:** Paul Manka, Lars P Bechmann, Frank Tacke, Jan-Peter Sowa, Martin Schlattjan, Julia Kälsch, Christoph Jochum, Andreas Paul, Fuat H Saner, Christian Trautwein, Guido Gerken, Ali Canbay

**Affiliations:** 1 Department of Gastroenterology and Hepatology, University Hospital, University Duisburg-Essen, Hufelandstr 55, Essen, 45122, Germany; 2 Department of Medicine III (Gastroenterology and Metabolic Disorders), University Hospital Aachen, Aachen, Germany; 3 Department of General, Visceral and Transplantation Surgery, University Hospital, University Duisburg-Essen, Essen, Germany

**Keywords:** ALF, MELD, MELD-Na, UKELD, Sodium

## Abstract

**Background:**

Acute liver failure (ALF) is a devastating clinical syndrome with a high mortality rate. The MELD score has been implied as a prognostic tool in ALF. Hyponatremia is associated with lethal outcome in ALF. Inclusion of serum sodium (Na) into the MELD score was found to improve its predictive value in cirrhotic patients. Therefore the aim of this study was to determine whether inclusion of serum Na improves the predictive value of MELD in ALF compared to established criteria.

**Methods:**

In a prospective single center study (11/2006–12/2010), we recruited 108 consecutive ALF patients (64% females / 36% males), who met the criteria defined by the “Acute Liver Failure Study Group Germany”. Upon admission, clinical and laboratory data were collected, King’s College Criteria (KCC), Model of End Stage Liver Disease score (MELD), and serum sodium based modifications like the MELD-Na score and the United Kingdom Model of End Stage Liver Disease score (UKELD) were calculated and area under the receiver operating characteristic curve analyses were performed regarding the prediction of spontaneous recovery (SR) or non-spontaneous recovery (NSR; death or transplantation).

**Results:**

Serum bilirubin was of no prognostic value in ALF, and Na also failed to predict NSR in ALF. The classical MELD score was superior to sodium-based modifications and KCC.

**Conclusions:**

We validated the prognostic value of MELD-Na and UKELD in ALF. Classic MELD score calculations performed superior to KCC in the prediction of NSR. Serum Na and Na-based modifications of MELD did not further improve its prognostic value.

## Background

Acute liver failure (ALF) is a potentially lethal clinical syndrome with a high mortality rate. Nonetheless, immediate intensive care, specific therapies and liver transplantation (LTx) have improved the prognosis of ALF patients significantly [1-3]. According to the European Liver Transplant registry, approximately nine percent of LTx were related to ALF [4]. King’s college (KCC) and Clichy criteria are etiology specific, prognosis predicting allocation tools to warrant timely transplantation and fair organ distribution [5,6]. However, their accuracy to reliable predict patients’ individual prognosis and to discriminate those patients who will survive without LTx remains a major challenge [7,8].

The model of end-stage liver disease (MELD), initially established to predict survival following transjugular intrahepatic portosystemic shunt (TIPS) procedure and later as an allocation tool for patients with cirrhosis, has been implied as a prognostic tool in ALF and was proven to be superior to the KCC and Clichy criteria [9-11]. Recently, various modifications of the MELD have been introduced and improved accuracy in both, chronic liver failure and ALF [12,13]. Since hepatic encephalopathy (HE) is associated with a fulminant course of ALF and a decrease in extracellular fluid osmolality is associated with an increase in brain swelling, factors that modulate fluid osmolality were taken into account as prognostic markers [14,15]. Patients with serum sodium between 145 and 150 mmol/l are known to have fewer episodes of intracranial hypertension and consequently a higher risk to develop brain edema. Thus, hyponatremia might worsen the prognosis in ALF [16]. As hyponatremia is associated with poor prognosis in cirrhosis, inclusion of serum sodium (Na) into the MELD was found to improve its predictive value in chronic liver diseases [13,17]. Two sodium containing MELD modifications, “UKELD” and “MELD-Na”, were proposed to enhance its prognostic ability in chronic liver failure [18,19]. A potential predictive value of these modifications in ALF has not been evaluated yet.

The aim of this study was to determine whether inclusion of serum sodium into the MELD score improves its predictive value in ALF, compared to established criteria. By evaluating these tools in a large prospective single-center study with ALF patients, we demonstrate that the sodium based MELD modifications do not improve the prognostic value of the standard MELD formula.

## Methods

### Patients and ethical considerations

The study was carried out according to the Declaration of Helsinki and the guidelines of the International Conference for Harmonization for Good Clinical Practice, it was approved by the local Ethics Committee of the University Hospital Essen (Institutional Review Board). In a prospective monocenter study (11/2006–12/2010), we recruited 108 consecutive ALF patients (64% females / 36% males), who met the criteria defined by the “Acute Liver Failure Study Group Germany” [20]. In brief, ALF was diagnosed by significant liver dysfunction with pathologically increased laboratory parameters (bilirubin, AST, ALT, AP, γ-GT) and an international normalized ratio (INR) of >1.5 with the concomitant presence of any degree of encephalopathy. Reference values for normal ranges are presented in Table 1. A pathological increase was defined as any value above these ranges. Other causes of liver dysfunction were excluded, such as acute-on-chronic liver failure or pre-existing cirrhosis. All patients had presented within four weeks of disease onset without pre-existing liver disease. Upon admission, clinical data were collected. Outcome (spontaneous recovery, SR; non-spontaneous recovery, NSR: comprising transplantation or death) was defined by the status after 4 weeks post admission.

**Table 1:** Patient’s general characteristics and laboratory parameter by outcome

	**Reference ranges**	**Spontaneously recovered (SR) (n=65)**	**Not spontaneously recovered (NSR) (n=43)**	**Transplanted ( LTx) (n=24)**	**Deceased (†) (n=19)**	**SR vs. NSR**	**SR vs. †**	**SR vs. LTx**	**LTx vs. †**
**Gender female (%)**		38 (58%)	31 (72%)	17 (71%)	14 (73%)				
Age**		41 (64)	49 (61)	41.5 (48)	56 (54)	p<0.05	p<0.05	n.s.	p<0.05
**BMI (kg/m**^**2**^) **		23.37 (30.59)	26.64 (46.83)	25.91 (28.54)	27.7 (42.82)	n.s.	n.s.	n.s.	n.s.
PLATELETS [/nl]**	140-380	181 (350)	165 (450)	176.5 (450)	130 (325)	n.s.	n.s.	n.s.	n.s.
GGT [U/l]**	<55	113 (1102)	154 (1938)	154 (720)	158 (1938)	n.s.	n.s.	n.s.	n.s.
ALP [U/l]**	25-124	151 (3024)	183 (670)	173 (450)	214 (571)	n.s.	p<0.05	n.s.	n.s.
AST [U/l]**	<50	2329 (18650)	1081 (15388)	837 (4642)	2342 (15388)	n.s.	n.s.	p<0.05	n.s.
ALT [U/l]**	<50	2288 (12955)	1092 (7954)	946.5 (7177)	1682 (7835)	p<0.05	n.s.	p<0.05	n.s.
INR**	0.89-1.11	1.77 (2.96)	3.35 (8.36)	3.64 (7.88)	2.88 (8.17)	p<0.05.	p<0.05	p<0.05	n.s.
Direct bilirubin [mg/dl]**	<0.2	5.8 (28.7)	13.8 (30)	18.25 (28)	9.3 (24.4)	p<0.05	n.s.	p<0.05	p<0.05
Total bilirubin [mg/dl]**	0.3-1.2	11.4 (40.5)	20.2 (43.1)	21.85 (37.7)	15.4 (38.9)	p<0.05	n.s.	p<0.05	p<0.05
Creatinine [mg/dl]**	0.6-1.3	0.99 (5.09)	1.81 (4.43)	1.385 (4.43)	2.5 (3.87)	p<0.05	p<0.05	p<0.05	p<0.05
Sodium [mmol/l]**	136-145	138 (24)	138 (27)	137.5 (23)	139 (27)	n.s.	n.s.	p<0.05	n.s.
MELD*		23.55 ± 0.66	36.37 ± 0.68	36.33 ± 0.8638	36.42 ± 1.12	p<0.05	p<0.05	p<0.05	n.s.
UKELD*		58.39 ± 0.57	65.12 ± 0.73	66.29 ± 0.8666	63.47 ± 1.20	p<0.05	p<0.05	p<0.05	n.s.
MELD-Na*		24.5 ± 0.65	36.49 ± 0.65	36.67 ± 0.81	36.24 ± 1.1	p<0.05	p<0.05	p<0.05	n.s.

*Mean ± Standard Error; *p<0.05 (Student’s t-Test for unrelated groups; SPSS software, version).**Median (Range); **p<0.05 (Mann- Whitney- U- Test; SPSS software).

### Assessment of prognosis

Serum sodium, KCC, MELD, UKELD and MELD-Na were assessed upon admission. Parameters were correlated with the outcome at 4 weeks after admission. In order to calculate the individual MELD score we used the formula as published by Kamath *et al.*[21]. If the bilirubin and creatinine values were below 1.0 mg/dl they were set to 1.0 mg/dl. If the creatinine was above 4.0 mg/dl or the patients underwent dialysis during two weeks before assessment, the creatinine value was adjusted to 4.0 mg/dl. In order to calculate the individual MELD-Na score we used the formula as published by Ruf *et al.*[17]. If the sodium values were below 125 mmol/l they were set to 125 mmol/l, if the values were above 140 mmol/l they were adjusted to 140 mmol/l. In order to calculate the individual UKELD score we used the formula as published by Barber *et al.*[19]. The KCC were evaluated following published criteria. Differentiation between acetaminophen induced ALF and non-acetaminophen induced ALF have been made, as previously published [22]. Detailed formulas of the utilized scores are given in Table 2.

**Table 2:** Model of end-stage liver disease (MELD) formula and sodium dependent modifications

	
**Score**	**Formula**
MELD	$\mathrm{MELD}=10\;\left\{\begin{array}{c} 0.957\;\ln\left(\mathrm{creatinine}\;\left[\mathrm{mg}/\mathrm{dl}\right]\right)\\ +0.378\;\ln\left(\mathrm{total}\;\mathrm{bilirubin}\;\left[\mathrm{mg}/\mathrm{dl}\right]\right)\;\\ +1.12\;\ln\left(\mathrm{INR}\right)+0.643 \end{array}\right\}$
MELDNa	*MELDNa* = *MELD* − *Na* − [0.025 × *MELD* × (140 − *Na*)] + 140
UKELD	$\mathrm{UKELD}=\left\{\begin{array}{c} \left(5.395\mathrm{xln}\left(\mathrm{INR}\right)\right)\;\\ +\left(1.485\mathrm{xln}\left(\mathrm{creatinine}\;\left[\mathrm{\mu mol}/l\right]\right)\right)\;\\ +\left(3.13\mathrm{xln}\left(\mathrm{total}\;\mathrm{bilirubin}\;\left[\mathrm{\mu mol}/l\right]\right)\right)\\ –\left(81.565\mathrm{xln}\left(\mathrm{Sodium}\;\left[\mathrm{mmol}/l\right]\right)\right) \end{array}\right\}+435$

### Statistics

Differences between parameters were evaluated by one-way Analysis of Variance, repeated-measure Analysis of Variance, or paired Student’s t-test and t-test for independent samples t-test. For MELD and modified MELD statistics the Mann–Whitney test was used. For categorical variables, frequencies and percentages were estimated. χ^2^ or Fisher’s exact tests were used for categorical factors. ROC calculations were undertaken where applicable. Screening, optimal, and diagnostic cutoff values were calculated, and the optimal cutoff, including specificity and sensitivity as well as the AUC are shown in boxes included in the ROC plots. A *p* < 0.05 was considered statistically significant. All values are given as means ± standard error of means. Analyses were performed with SPSS 19.0.1, version 2008 (SPSS, Chicago, IL, USA).

## Results

### Demographic data

**Figure 1:**
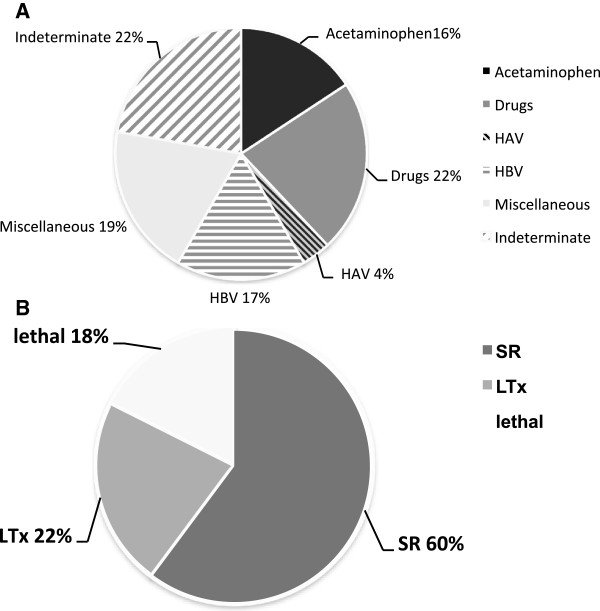
**Etiologies and Outcome of patients with ALF. A**) Drug toxicity was the most frequent single cause ALF with acetaminophen intoxication representing 17 cases (16%) in this prospective cohort (n=108). Viral causes were responsible for 22 of all cases with ALF, with HBV infection representing 18 cases. 21 ALF cases were due to miscellaneous causes (Wilson’s disease, Amanita intoxication, Epstein-Barr-Virus-Infection). In 24 cases the underlying etiology remained indeterminate. **B**) Most patients recovered spontaneously from ALF (SR; n=65), 19 patients died without transplantation and 24 were transplanted.

Mean age of the 108 patients [69 (64%) females and 39 (36%) males] was 43.4 ± 16.2 Underlying etiologies are presented in Figure 1A. Detailed laboratory parameters and general data are listed in Table 1. Sixty-two (60%) patients recovered spontaneously (SR), fifty-four (40%) deceased or underwent LTx (NSR) (Figure 1B). Differences concerning outcome in patients with non-acetaminophen induced ALF are presented in Table 3.

**Table 3:** Patient’s general characteristics and laboratory parameter by outcome in cases of non-acetaminophen induced acute liver failure

		**INR**	**Bilirubin [mg/dl]**	**Creatinine [mg/dl]**	**Sodium [mmol/l]**	**MELD**	**UKELD**	**MELD-Na**
**SR**	**N**	**50**	**50**	**50**	**49**	**50**	**49**	**49**
	Mean	1.8	14.6	1.34	138.20	23.92	58.85	24.82
	Standart deviation	0.45	9.44	1.19	4.41	4.87	4.52	4.86
	Standar error	0.07	1.33	0.17	0.63	0.69	0.65	0.69
	Median	1.74	15.65	0.99	138.00	24.00	60	25
	Skewness	1.02	0.24	2.38	−0.202	−0.126	−0.714	−0.137
**NSR**	**N**	**41**	**41**	**41**	**40**	**41**	**40**	**40**
	Mean	3.98	20.8195	2.16	138.6	36.24	65.1	36.45
	Standart deviation	2.12	10.92050	1.22595	6.41233	4.53200	4.75988	4.19982
	Standard error	0.33	1.71	0.19	1.01	0.71	0.75	0.66
	Median	3.35	20.2	1.81	138	38	66	38
	Skewness	1.56	0.43	0.56	0.89	−0.79	−0.61	−0.92
**Deceased**	**N**	**18**	**18**	**18**	**17**	**19**	**17**	**17**
	Mean	4	16.67	2.53	140.29	36.22	63.47	36.24
	Standart deviation	2.292	10.53	1.23	7.48	4.93	4.96	4.53
	Standard error	0.54	2.48	0.29	1.81	1.16	1.20	1.1
	Median	3.12	14.85	2.45	139	39	63	38
	Skewness	1.451	0.859	0.221	0.555	−0.916	−0.194	−0.985
**SR vs. NSR**		P<0.01**	P<0.01*	P<0.01*	n.s. p=0.732	P<0.01*	P<0.01*	P<0.01*
**SR vs. Deceased**		P<0.01**	n.s.	P<0.01*	n.s. p=0.169	P<0.01*	P<0.01*	P<0.01*

*p<0.05 (Student’s t-Test for unrelated groups; SPSS software, version).

**p<0.05 (Mann- Whitney- U- Test; SPSS software).

### Sodium levels fail to predict outcome in ALF

**Figure 2:**
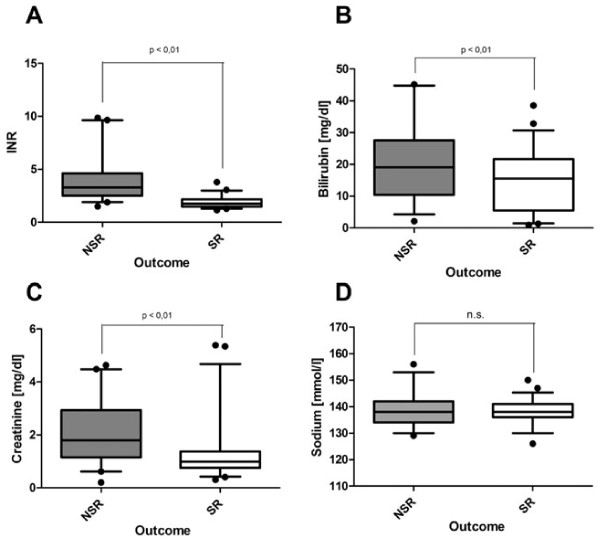
**Serum markers of liver damage and serum sodium in non-acetaminophen induced ALF.** Standard serum parameters of liver injury were assessed during diagnostic procedures in ALF patients, to determine status and underlying etiology. Recordings are depicted for the day of maximum MELD score. (**A**) INR; (**B**) bilirubin; (**C**) creatinine; (**D**) sodium. All data are depicted as means ± SEM. SR: spontaneous recovery, NSR: non-spontaneous recovery, *: *p* vs. NSR <0.05.

**Figure 3:**
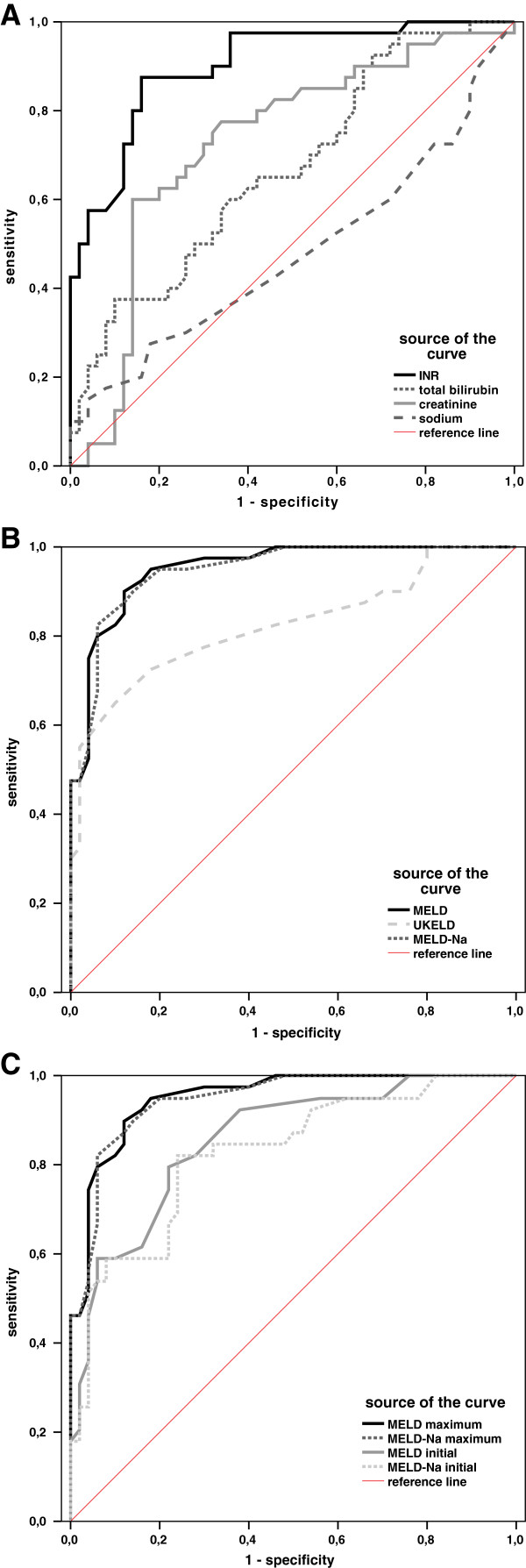
**ROC curves for single parameters of the MELD, sodium and different predictive scores in non-acetaminophen induced ALF.** The prognostic value of single parameters comprising the classical MELD score was compared to serum sodium. The ROC curve shows the prognostic value of INR, creatinine, bilirubin and serum sodium at the point of maximum MELD during 4 weeks after admission (**A**). In addition the prognostic value of the classic MELD score was compared to sodium-based MELD modifications (UKELD and MELD-Na) and the INR as best single prognostic parameter as reference. Classical MELD provides the best performance of the given scores (**B**). Differences in the predictive value between the MELD and MELD-Na at the time of admission and at the time-point of its biggest dimension (**C**).

To assess the predictive value on the clinical outcome of sodium in comparison to individual MELD parameters, we analyzed the individual parameters at the date of the maximum MELD score within 4 weeks after admission. Upon the point of maximum MELD, significant differences between SR and NSR groups were found for all of the individual MELD parameters, including INR (SR 1.9 ± 0.08, NSR 3.99 ± 0.32, *p* < 0.05; Figure 2A), total bilirubin (SR: Median: 11.4 Range: 40.5 [mg/dl], NSR: Median: 20.2; Range: 43.1 Figure 2B) and creatinine (SR 1.32 ± 0.14 mg/dl, NSR 2.15 ± 0.19 mg/dl, *p* < 0.05; Figure 2C). In contrast to these findings, serum sodium did not show any difference between these two groups (SR 138.63 ± 0.57 mmol/l, NSR 138.63 ± 0.99 mmol/l; Figure 2D). Analyses of the performance for the individual MELD parameters (Figure 3A) showed that increased INR at date of maximum MELD had a modest sensitivity and specificity (both 88%) (INR: cutoff: 2.47, AUC: 0.922; 95% CI: 0.867-0.977; *p* < 0.05). Increased creatinine alone had a sensitivity of 75% and a specificity of 70% at a cutoff value of 1.16 mg/dl (creatinine: AUC 0.723; 95% CI: 0.629-0.846; *p* < 0.05). In contrast, serum bilirubin lacked specificity at date of maximum MELD. However, a cutoff for serum bilirubin was calculated with an anticipated low specificity (bilirubin: cutoff: 19.8 mg/dl; AUC 0.661; 95% CI 0.54-0.774; *p* < 0.05). ROC curve analysis for serum sodium revealed no significant prognostic value.

### Sodium based modifications fail to improve MELD in ALF

The classical MELD score showed a strong correlation with the clinical outcome (Figure 3B: MELD: AUC 0.967; CI 0.935 – 0.998; *p* < 0.01). Since hyponatremia is associated with an unfavorable prognosis, inclusion of serum sodium into the MELD score was found to improve its predictive value in cirrhotic patients [13,17]. However, in our ALF cohort, the classical MELD was superior to sodium based modifications like the MELD-Na (AUC: 0.960; CI: 0.924-0.966; *p* < 0.01) or the UKELD (AUC: 0.828; CI: 0.738-0.918; *p* < 0.01).

### MELD and MELD-Na are more sensitive than KCC in predicting NSR in non-acetaminophen induced ALF

A comparison of the MELD score, MELD-Na and the KCC in non-acetaminophen induced ALF demonstrated a better performance of the MELD based scores (Table 4). Concerning sensitivity, specificity, negative predictive value (NPV), positive predictive value (PPV) and overall diagnostic accuracy, the classic MELD was superior to KCC (MELD: Sensitivity: 0.90, Specificity: 0.92, PPV: 0.90, NPV: 0.92, diagnostic accuracy: 0.912; KCC: Sensitivity: 0.72, Specificity: 0.90, PPV: 0.86, NPV: 0.79, diagnositc accuracy: 0.818). While the accuracy of MELD-Na to predict outcome was lower, compared to classic MELD score, MELD-Na performed still better than KCC (MELD-Na: Sensitivity: 0.90, Specificity: 0.90, PPV: 0.88, NPV: 0.92 and diagnostic accuracy: 0.898).

**Table 4:** Comparison of the predictive values, sensitivity, specificity and diagnostic accuracy of the King’s College Criteria, MELD and MELD-Na at the date of maximum MELD during four weeks after admission and the MELD at date of admission

**Score**	**Sensitivity**	**Specificity**	**PPV**	**NPV**	**Diagnostic accuracy**
**Maximum MELD**	0.90	0.92	0.90	0.92	0.912
**KCC**	0.72	0.90	0.86	0.79	0.818
**MELD-Na**	0.90	0.90	0.88	0.92	0.898
**Initial MELD**	0,78	0,76	0.73	0.81	0.772
**Initial MELD-Na**	0,80	0,76	0,72	0,82	0,780

### Prediction of lethal outcome for MELD and its sodium modifications depends on the extent of liver injury

A comparison of the MELD score at time of admission and the timepoint with a maximum MELD score within four weeks after admission in non-acetaminophen induced ALF revealed a better performance of the “maximum MELD” based scores (Table 4). Concerning sensitivity, specificity, negative predictive value (NPV), positive predictive value (PPV) and overall diagnostic accuracy the maximum MELD values were superior to the initial ones (initial MELD: AUC: 0.839; CI: 0.757-0.922; *p* < 0.01 Sensitivity: 0.78, Specificity: 0.76, PPV: 0.73, NPV: 0.81, diagnostic accuracy: 0.772, initial MELD-Na: AUC: 0.825; CI: 0.738-0.913, p<0.01 Sensitivity: 0.80, Specificity: 0.76, PPV: 0.72, NPV: 0.83, diagnostic accuracy 0.780).

## Discussion and conclusion

To our knowledge, this is the first study to evaluate a potential predictive role for serum sodium based MELD modifications in the clinical setting of ALF in a large prospective cohort. Compared to the individual MELD parameters and in contrast to patients with chronic liver disease or post-transplant outcomes for acute liver failure [23], we could not find a clear association between serum sodium levels and clinical outcome in ALF which is in line with recent studies [24]. Accordingly, the serum sodium based MELD modifications MELD-Na and UKELD failed to improve the predictive value of the MELD in ALF patients. However, in our cohort the classic MELD as well as MELD-Na was superior to KCC in predicting outcome of ALF patients. Interestingly, hypernatremia was associated with lethal outcome in our ALF cohort. In contrast in chronic liver disease hyponatremia is associated with a worse outcome even for mid to long term survival [25], the rational for utilizing UKELD and MELD-Na in cirrhosis.

Several studies have demonstrated a good specificity for KCC in ALF, however, the sensitivity to predict lethal outcome was modest in the vast majority of studies, especially in non-acetaminophen induced ALF [2,6,26]. Here, we found a fairly good specificity for KCC to predict NSR and as previously published only a modest sensitivity. Recent studies identified MELD as a prognostic tool with better sensitivity compared to KCC [21,27]. Specifically, in patients with non-acetaminophen induced ALF, MELD was superior to KCC in predicting outcome [9]. This is in line with our findings, which show a better performance of MELD and MELD-Na compared to KCC in non-acetaminophen ALF.

MELD was primarily introduced as a prognostic tool for survival of patients with cirrhosis and portal hypertension following TIPS procedure [28]. Later, it was found to be useful in organ allocation for patients with chronic liver disease awaiting liver transplant and is therefore widely used in Western societies [21]. As hyponatremia is a common clinical problem in patients with end stage liver disease, especially in those individuals with portal hypertension, ascites and hepatorenal syndrome, sodium based modifications of the MELD have been introduced. In the UK, the UKELD is utilized instead of the MELD for organ allocation [19]. Murphy *et al.* identified hyponatremia as an independent risk factor for brain edema, a fatal complication of ALF [16,29]. Furthermore hyponatremia has been investigated extensively in the management of traumatic cerebral edema [30,31]

In our study we could not find an advantage of sodium based MELD modifications, compared to MELD or KCC in ALF. This is most likely due to the absence of ascites or hepatorenal syndrome in ALF, both common co-morbidities of cirrhosis [32,33]. In our cohort, we did not find a difference in sodium levels between SR and NSR. Although Murphy *et al*. showed that patients with serum sodium between 145 and 150 mmol/l had fewer episodes of intracranial hypertension. There has been no difference in outcome [16]. Taken together we confirm previous publications, establishing MELD as a powerful prognostic tool for non-acetaminophen induced ALF patients.

Furthermore, our data revealed a crucial problem with the assessment of any prognostic factor. We found significant differences in its predictive value between the MELD at the time of admission and at the time-point of its biggest dimension. The maximum MELD performed best in predicting outcome in ALF, underlining the need for continuous clinical assessment of patients with ALF, given the heterogeneity and dynamic of this disease. However, this also shows the need for novel prognostic models and surrogate parameters for the degree of liver injury and disease progression [34]. While other possible MELD modifications might improve its accuracy in the future [12], sodium based MELD modifications are of little prognostic value in the clinical setting of ALF. Remien et al., in contrast to other modifications of the KCC and MELD-Score, developed the MALD score which is novel as it builds upon the KCC by utilizing an understanding of the dynamics of hepatocyte damage following APAP overdose in the form of a dynamic mathematical model [35]. As ALF is a devasting clinical condition, it is worth it to evaluate new prognostic tools to improve outcome in this patients.

## Abbreviations

ALF: Acute liver failure; KCC: Kings college criteria; MELD: Model of end-stage liver disease; Na: Sodium; NPV: Negative predicte value; NSR: No spontaneous remission; PPV: Positive predictive value; SR: Spontaneous remission; TIPS: Transjugular intraheptic portosystemic shunt.

## Competing interests

The authors declare that they have no competing interest.

## Authors’ contributions

PM participated in the study design, collected patient material, performed statistical analyses and drafted the manuscript. LPB collected patient material and revised the manuscript for important intellectual content. FT participated in the study design and revised the manuscript for important intellectual content. JPS performed statistical analyses and revised the manuscript for important intellectual content. MS performed detection of various serum parameters. JK collected patient material. CJ participated in the study design and coordination and collected patient material. AP participated in study coordination and collected patient material. FHS participated in study coordination and collected patient material. CT participated in the study design and revised the manuscript for important intellectual content. GG participated in study coordination and revised the manuscript for important intellectual content. AC conceived of the study, and participated in its design and coordination and revised the manuscript for important intellectual content. All authors read and approved the final manuscript.

## Pre-publication history

The pre-publication history for this paper can be accessed here:

http://www.biomedcentral.com/1471-230X/13/58/prepub
